# Antimicrobial Peptide TP4 Targets Mitochondrial Adenine Nucleotide Translocator 2

**DOI:** 10.3390/md18080417

**Published:** 2020-08-09

**Authors:** Bor-Chyuan Su, Yi-Chung Liu, Chen-Hung Ting, Ping-Chiang Lyu, Jyh-Yih Chen

**Affiliations:** 1 Department of Anatomy and Cell Biology, School of Medicine, College of Medicine, Taipei Medical University, Taipei 110301, Taiwan; su8265@gmail.com; 2 Institute of Population Sciences, National Health Research Institutes, 35 Keyan Rd., Zhunan, Miaoli County 350, Taiwan; jong212@gmail.com; 3 Marine Research Station, Institute of Cellular and Organismic Biology, Academia Sinica, 23-10 Dahuen Rd., Jiaushi, Ilan 262, Taiwan; koichiting@gmail.com; 4 Institute of Bioinformatics and Structural Biology, National Tsing-Hua University, 101, Sec. 2, Kuang-Fu Rd., Hsinchu 300, Taiwan; pclyu@mx.nthu.edu.tw; 5 The iEGG and Animal Biotechnology Center, National Chung Hsing University, Taichung 402, Taiwan

**Keywords:** antimicrobial peptide (AMP), tilapia piscidin 4 (TP4), adenine nucleotide translocator 2 (ANT2)

## Abstract

Tilapia piscidin (TP) 4 is an antimicrobial peptide derived from Nile tilapia (*Oreochromis niloticus*), which shows broad-spectrum antibacterial activity and excellent cancer-killing ability in vitro and in vivo. Like many other antimicrobial peptides, TP4 treatment causes mitochondrial toxicity in cancer cells. However, the molecular mechanisms underlying TP4 targeting of mitochondria remain unclear. In this study, we used a pull-down assay on A549 cell lysates combined with LC-MS/MS to discover that TP4 targets adenine nucleotide translocator (ANT) 2, a protein essential for adenine nucleotide exchange across the inner membrane. We further showed that TP4 accumulates in mitochondria and colocalizes with ANT2. Moreover, molecular docking studies showed that the interaction requires Phe1, Ile2, His3, His4, Ser11, Lys14, His17, Arg21, Arg24 and Arg25 residues in TP4 and key residues within the cavity of ANT2. These findings suggest a mechanism by which TP4 may induce mitochondrial dysfunction to disrupt cellular energy metabolism.

## 1. Introduction

Antimicrobial peptides (AMPs) are components of the innate immunity response, wherein they function to combat pathogen infection by mechanisms that are conserved across species [1,2]. The protective mechanisms of individual AMPs are derived from their characteristic structural properties, such as an overall positive charge. Another common property of these peptides is amphiphilicity. This property allows many AMPs to target pathogens via electrostatic interactions, which stimulate membrane pore formation according to the toroidal, barrel-stave or carpet models, and finally result in cell lysis [3,4,5]. Moreover, other non-lytic pathogen-killing pathways, which involve the targeting of specific cellular factors, have also been reported [6]. Interestingly, some pathogen-killing activities may also allow AMPs to target cancer cells, and several AMPs have been implicated as promising anticancer agents for various cancers [7,8,9,10,11,12]. One such AMP, Tilapia piscidin (TP) 4, was identified in Nile tilapia (*Oreochromis niloticus*) [13]. A growing body of literature has shown that a synthesized active segment of TP4 has broad-spectrum antibacterial activities [13,14,15] as well as excellent cancer cell-killing activity in different cancer types in vitro and in vivo [9,10,11,12,16,17]. Multiple anticancer mechanisms are thought to be involved in AMP-mediated cytotoxicity, including rapid cell membrane disruption at high-dose treatment [18,19]. On the other hand, low-dose AMP treatment may selectively target cancer cells based on the charge of the cell membrane [11,17,18]. The negatively charged plasma membrane of cancer cells attracts cationic AMPs through electrostatic interactions. Once bound to the cancer cells, some AMPs exert cytotoxicity simply through membrane lysis, thus preventing any robust development of multiple-drug resistance (MDR) [18,20]. However, other AMPs penetrate the cancer cells and target different organelles [10,11,12,16,19,21,22]. These actions of membrane lysis and organelle targeting can trigger either necrotic or apoptotic pathways in different cancer cell lines [18]. We have previously shown that penetration of fish-derived AMPs induces cellular stress and activates FBJ osteosarcoma oncogene (FOS) family transcription factors to initiate necrotic cell death [11,12,17]. The activation of FOS family members was found to be caused by dysregulation of Ca^2+^ homeostasis, as elimination of Ca^2+^ signaling by a chelator largely reduced FOS family activation and AMP-induced necrotic death [11,12,16,17,19]. In addition, disruption of the cytoskeleton network was observed in AMP-treated cancer cells [10,12,23,24]. In this context, tubulin was shown to interact with TP4 by co-immunoprecipitation (IP) and liquid chromatography-tandem mass spectrometry (LC-MS/MS) approaches [10]. Thus, cytoskeletal dysregulation appears to be a major contributor to AMP-mediated anti-cancer activities. Interestingly, mitochondrial toxicity, involving dysregulated Ca^2+^ homeostasis, elevated reactive oxygen species and loss of membrane potential, has also been found to serve as a common determinant of AMP-induced cancer cell death [8,11,12,16,17,21,22,25]. These studies suggest that in addition to cytoskeletal proteins, mitochondria may also be a primary intracellular target of these molecules. Although mitochondrial damage is clearly caused by AMPs [11,12,16], it is unknown whether any specific mitochondrial protein might be targeted by AMPs and what cytotoxic mechanisms such targeting would invoke.

The adenine nucleotide translocators (ANTs) (also called ADP/ATP translocases) belong to the mitochondrial carrier superfamily [26]. These proteins mediate the exchange of ADP/ATP across the mitochondrial inner membrane [27] and regulate the mitochondrial permeability transition pore [28]. Four ANT isoforms (ANT1 through ANT4, encoded by *SLC25A4*, *SLC25A5*, *SLC25A6*, and *SLC25A31,* respectively) have been characterized in humans [29]. ANT1 is predominantly expressed in differentiated cells, such as heart and muscle [30], while ANT2 is expressed systemically and is inducible [31]. Low level but systemic expression of ANT3 was also reported [32], and ANT4 shows a testis-specific expression pattern [33]. ANT1 and ANT3 function to import cytosolic ADP and export the oxidative phosphorylation product, ATP, from the mitochondrial matrix [34]. ANT2, however, is thought to import cytosolic ATP (produced by glycolysis) and export ADP [34]. In addition, an inverse orientation of ANTs has been speculated to occur in the mitochondrial inner membrane [35]. This could provide an explanation for the finding that cancer cells with high ANT2 expression are resistant to ANT inhibitors that normally block ATP entry into mitochondria [36,37]. Overexpression of ANT1 or ANT3 induces apoptosis, suggesting a pro-apoptotic role for these proteins [38,39]. ANT2, by contrast, plays a crucial role in cancer metabolism [34]. Suppression of ANT2 expression in cancer cells inhibits tumor growth both in vitro and in vivo, indicating its anti-apoptotic ability [40,41,42,43]. Due to the fact that ANT2 is essential for highly proliferative cells and cancer, drugs that target mitochondrial ANT2 may be useful as cancer therapeutics.

In this study, we discovered that ANT2 is a cellular target of TP4 via antibody pull-down and LC-MS/MS. The accumulation of TP4 into mitochondria was validated by super-resolution confocal microscopy, and the molecular interaction between TP4 and ANT2 was further defined using computational molecular docking analysis.

## 2. Results

### 2.1. TP4 Interacts with ANT2

**Figure 1:**
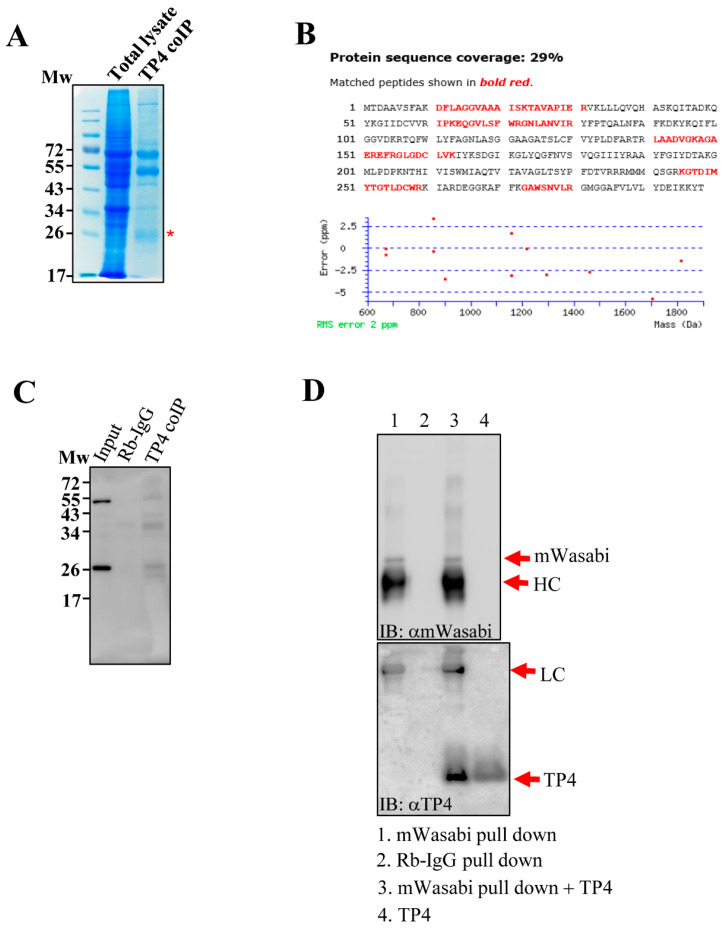
TP4 interacts with ANT2. (**A**) Coomassie blue-stained SDS-PAGE shows that a band around 26 kDa is co-immunoprecipitated by the TP4 antibody. Input lane was loaded with total lysate, before co-immunoprecipitation (IP). TP4-IP lane was loaded with IP from TP4 antibody. The protein band excised for in-gel digestion and LC-MS/MS analysis is labeled with a red asterisk (*). (**B**) A protein database search of peptides detected by MS revealed ANT2 as a potential TP4-interacting protein. ANT2 sequence is shown. Red letters denote peptides identified by the MS analysis. (**C**) Immunoblotting with an antibody against ANT2. Mw indicates molecular weight. (**D**) Monomeric wasabi (mWasabi)-tagged ANT2 was pulled down by an anti-mWasabi antibody (lane 1). Rabbit IgG pulldown was used as a negative control (lane 2). Pulled-down protein from the mWasabi pulldown group was incubated with TP4 (lane 3) and washed before immunoblotting with the TP4 antibody. TP4 alone served as a positive control (lane 4)**.**

In a previous study, we found that tubulin is an intracellular target of TP4 [10]. However, observations of specific mitochondrial targeting by TP4 [11,12] led us to speculate that the AMP may also target mitochondrial proteins. To test this hypothesis, cell lysates from TP4-treated A549 cells (10 µg/mL) were immunoprecipitated with an antibody against TP4. Pulled down proteins were analyzed by SDS-PAGE, and a major protein band at around 26 kDa was excised (Figure 1A). LC-MS/MS analysis of the band suggested that ANT2 was a candidate TP4-interacting protein (Figure 1B and Supplementary Data S1). Next, immunoprecipitated protein samples were probed with an ANT2 antibody, confirming that TP4 interacts with ANT2 in vivo (Figure 1C). In addition, A549 cells were made to overexpress mWasabi-tagged ANT2, and extracts were made from the cells. ANT2 was pulled down with a mWasabi antibody, followed by incubation of the pulled-down proteins with TP4. After washing, samples were analyzed by SDS-PAGE and Western blotting. The result showed that ANT2 interacts with TP4 in vitro (Figure 1D). Together, these results suggest that TP4 penetrates intracellularly and binds to the mitochondrial protein, ANT2.

### 2.2. Immunocytochical Study of TP4–ANT2 Interaction

**Figure 2:**
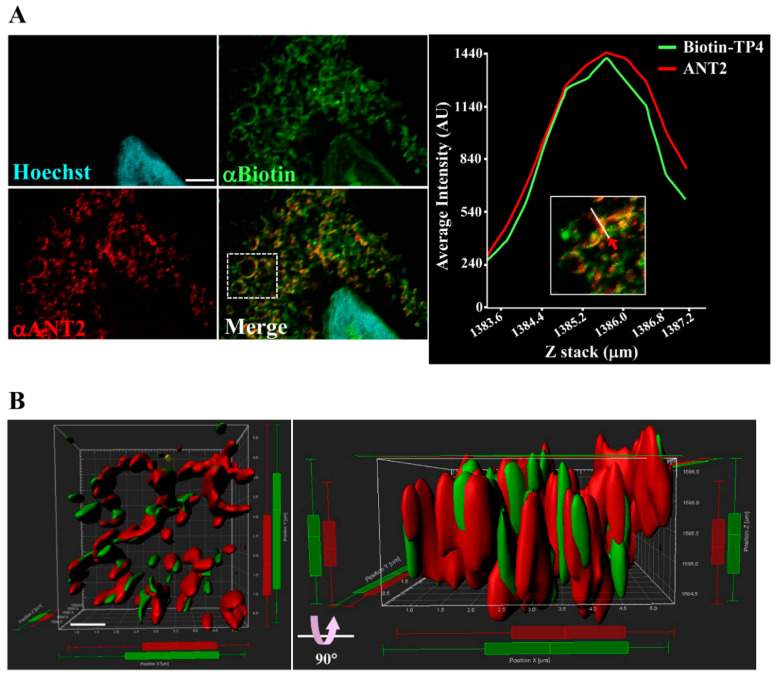
TP4 integrates into the mitochondria. (**A**) A549 cells pretreated with 10 µg biotinylated TP4 for 1 h at room temperature (RT) were fixed and stained for Biotin (green) and ANT2 (red). Hoechst33258 was used to stain the nucleus (cyan). Bar: 3 µm. Higher magnification of the boxed area in the merged panel is shown at the right side. The spatial correlation of TP4 with ANT2 in the indicated region (white line, indicated by red arrow) is shown by a line-series analysis. The green and red curves in the right panels represent the TP4 and ANT2 fluorescence intensities, respectively. AU: arbitrary units. (**B**) Three-dimensional integration of TP4 and ANT2 signals was simulated by Imaris software. The front-view and side-view are shown on the left and right sides, respectively. Bar: 1 µm.

To further examine the interaction between TP4 and ANT2, as well as their spatial association, colocalization was determined by super-resolution confocal microscopy (Figure 2A, left panel) and quantified with a line-series intensity correlation (Figure 2A, right panel). The three-dimensional (3D) deconvoluted confocal microscopy image was processed for 3D modeling with Imaris software and showed a pattern of TP4-mitochondrial integration (Figure 2B).

### 2.3. Molecular Structure Building and Validation

Since no X-ray crystallography or NMR structure of ANT2 is currently available, we constructed a theoretical structural model of the protein by a homology modeling approach. We used the human ANT2 protein sequence to query PSI-BLAST and identified similar sequences in the PDB database with resolved 3D structures to use as starting templates. The closest homologous sequence available in PDB was a mitochondrial ADP/ATP carrier in complex with carboxyatractyloside (PDB ID: 1OKC), which showed 89% sequence identity and 99% query coverage with an E value of 0. The target-template alignment file was generated using a Modeller script file (Supplementary Figure S1). Modeller calculates a 3D model of the target completely automatically, when using its “automodel” function (Video S1). All Modeller script files were executed using the Modeller command prompt. The software generated twenty-five different models by optimizing the objective function in Cartesian space. Three different energy scores, including molpdf, discrete optimized protein energy (DOPE) and GA341, were computed for each of the generated models and compared to select a principal conformational structure (Supplementary Table S1). The DOPE model score is designed specifically for selecting the best structure from a collection of models built by Modeller. Model 10 (ant2.B99990010.pdb) had the lowest DOPE score (−31639.03711) and was chosen as the best comparative model for energy minimization and further analyses. The selected model was further assessed for stereochemical quality using various online diagnostic tools. The Ramachandran plot obtained from the RAMPAGE server revealed that 97.3% of the residues in the predicted ANT2 model were in the favored region, 1.7% of the residues were in the allowed region and only 1.0% of residues were in the outlier region (Supplementary Figure S2). The PROCHECK module of the PDBSum server, was further used to validate the overall structure geometry of the predicted model, with 95.3% of residues accommodated in the most favored regions, 3.9% of residues in additional allowed regions, 0.8% residues in generously allowed regions and no residues in disallowed regions; the average G factor value was 0.25 (Supplementary Data S2). Moreover, the root-mean-square deviation (RMSD) between the predicted ANT2 model and template structure was 0.39Å. These results indicated that the predicted model quality had robust stereochemical features and was similar to the template structure. The ERRAT score value (96.55%) was better than the ideal score value (95%), suggesting the predicted model is not limited by resolution (Supplementary Figure S3).

Furthermore, an overall qualitative assessment was performed by ProSA analysis. The ProSA Z-score value is displayed in a plot containing the Z-scores of all experimentally determined protein structures in PDB. The Z-score value for the predicted model was −4.78 (Supplementary Figure S4), which is within the range observed for experimentally determined protein structures of similar size and indicates a good overall quality of the protein model.

### 2.4. Molecular Docking for the TP4–ANT2 Interaction

**Figure 3:**
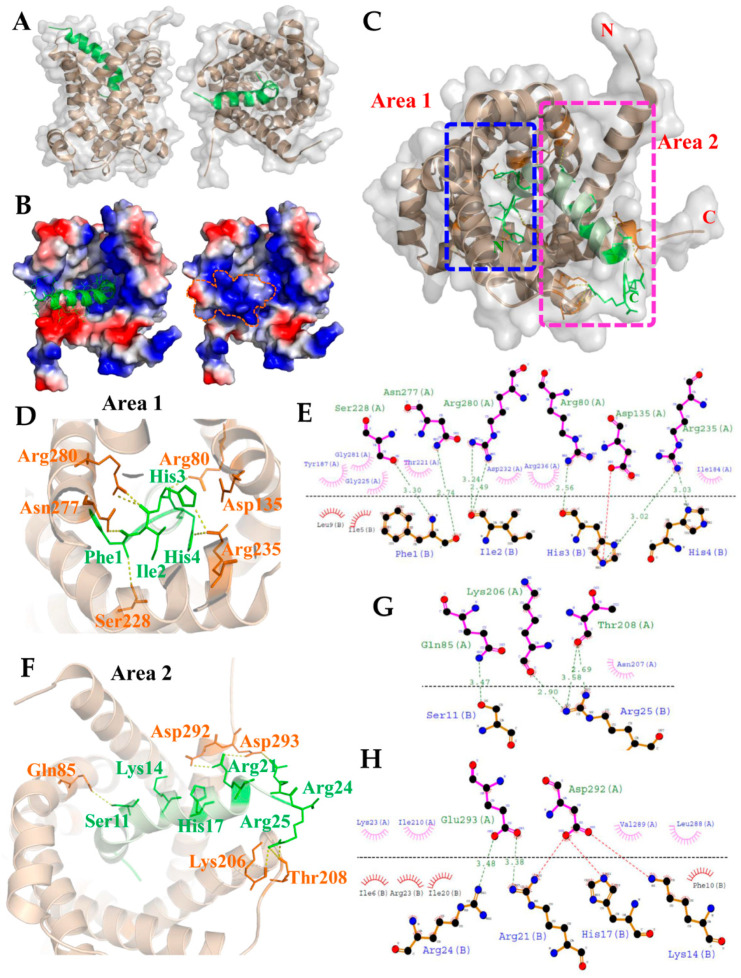
The model derived from molecular docking analysis of the TP4–ANT2 interaction. (**A**) The side and front views of the 3D interaction structure are shown for TP4 (green) and ANT2 (brown). (**B**) Electrostatic potential surface of the TP4–ANT2 complex is shown. Positive and negative charges are shown by blue and red colors, respectively. The N-terminal region of TP4 is bonded to the positively charged area at the bottom of the cavity in ANT2. (**C**) Two rectangular boxes (area 1 and area 2) indicate the polar interactions between TP4 and ANT2. Magnified images (**D**,**F**) show the relative positions of amino acids involved in polar interactions in area 1 and area 2, respectively. (**E**) LigPlot^+^ two-dimensional (2D) diagrams of the potential intermolecular interactions in area 1. (**G**,**H**) LigPlot^+^ 2D diagrams of the potential intermolecular interactions in area 2. Green dashed lines indicate hydrogen bonds. Red or pink eyebrow-like icons indicate hydrophobic interactions. Red dashed lines indicate salt bridges.

Since we showed that TP4 is integrated into the mitochondria and interacts with ANT2, we next evaluated the TP4–ANT2 interaction by a protein–protein docking method. The TP4 peptide structure and predicted ANT2 model were uploaded to the GRAMM-X docking server, which was used to perform a rigid body procedure. In the interface residue constraints fields, we restricted the receptor binding sites to the RRRMMM signature sequence from Arg235 to Met240, which is located at the bottom of the cavity and is expected to be involved in ADP/ATP binding [44]. All ADP/ATP carriers exhibit the RRRMMM consensus sequence, and this motif is highly conserved in ANTs across species [45]. The docking results showed that TP4 is likely to bind inside the cavity of ANT2 (Figure 3A). In the ANT2 structure, the bottom of the cavity is decorated with positively charged residues, as visualized by electrostatic surface potential calculations (Figure 3B). The N-terminal residues of TP4 bonded to the positively charged area at the bottom of the cavity. At the same time, the positively charged residues at the C-terminal end of TP4 bound to a negatively charged area at the wide-open hole of the cavity. In Figure 3C, two rectangular boxes (Area 1 and 2) indicate the polar interactions between ANT2 and TP4. Peptide–protein interaction analysis of the docked complex using PyMOL and Dimplot showed that the interface between ANT2 (Chain A) and TP4 (Chain B) is stabilized by hydrogen bonding, hydrophobic interactions and a salt bridge. The N-terminal region (Phe1, Ile2, His3, His4) of TP4 bound tightly to the bottom of the cavity, with hydrogen bonds between TP4 and ANT2 at: Phe1 with Ser228/Asn277; Ile2 with Arg280; His3 with Arg80/Arg235; His4 with Arg235 (Figure 3D). From the Dimplot analysis, around 13 residues of ANT2 at the bottom of the cavity are expected to participate in hydrophobic interactions with six residues of TP4. Additionally, one single salt bridge is likely to be formed between His3 of TP4 and Asp135 of ANT2 (Figure 3E). In the C-terminal region, hydrogen bonds will be formed between TP4 to ANT2 at: Ser11 with Gln85; Arg21 with Glu293; Arg24 with Glu293; Arg25 with Lys206/Thr208 (Figure 3F,G). From the Dimplot analysis, around 10 residues of ANT2 are expected to be involved in hydrophobic interactions with 10 residues of TP4 at the wide-open hole of the cavity. Moreover, salt bridges may be formed between Lys14, His17, Arg21 and Arg24 of TP4, and Asp292 and Glu293 of ANT2 to strengthen bonding (Figure 3H). The positive charge of the TP4 C-terminal region and the negative charge of ANT2 Asp292/Glu293 may further contribute to electrostatic and steric stabilization (Figure 3H).

## 3. Discussion

In this work, we identify ANT2 as a novel cellular target for TP4. We found that TP4 integrates into mitochondria and has a direct interaction with ANT2. Molecular docking analysis identified potentially critical amino acids in TP4 that are required for the interaction with ANT2, including Phe1, Ile2, His3, His4, Ser11, Lys14, His17, Arg21, Arg24 and Arg25.

Genetic studies on *Saccharomyces cerevisiae* ADP/ATP carrier isoform 2 (ScAnc2p) suggested several crucial amino acid residues required for ANT binding and transporter activities [46]. Interestingly, many of these amino acids may also be essential for targeting by TP4. ANTs belong to the mitochondrial carrier family of proteins, which shares highly similar structural features. For example, a tripartite element is organized in three sequence repeats, and each repeat contains a conserved motif as follows: P×(D/E)××(K/R)×(K/R)-(20–30 residues)-(D/E)G××××A_r_(K/R)G, where ″×″ represents other residues and “A_r_” denotes an aromatic residue [47]. In addition, a transport-enabling nucleotide carrier signature motif, RRRMMM, is commonly observed in ADP/ATP carrier proteins and localized at the third repeat [48]. The N-terminal region of the TP4 is predicted to bind tightly to the bottom of the cavity in our modelled ANT2 structure; this interaction is stabilized by hydrogen bonding, hydrophobic interactions and a salt bridge. In particular, Arg235 of ANT2 contributes the hydrogen bonding and hydrophobic interactions with His3 and His4 of TP4, while Arg236 of ANT2 also contributes hydrophobic interactions with Ile2 and His3 of TP4 in the RRRMMM motif of the docked complex (Figure 3E). Furthermore, three residues of ANT2, including Arg80, Asn277 and Arg280, provide important hydrogen bonding and hydrophobic interactions with Phe1, Ile2 and His3 of TP4. Notably, these residues were present in peptides identified by our LC-MS/MS analysis (Figure 1B).

In yeast, mutation of Lys38 in ScAnc2p (corresponding to Lys23 of ANT2, Figure 3G) largely inactivates ADP/ATP carrier function and impairs growth of the cells on non-fermentable carbon sources (e.g., glycerol) [49,50]. In addition, Arg80 of ANT2 (Figure 3E) corresponds to Arg96 in ScAnc2p, the mutation of which affects nucleotide exchange [51,52,53]. Moreover, a naturally occurring *pet9* Arg96His mutant of ScAnc2p lacks a functional ADP-ATP carrier, and exhibits defective respiration and failure to grow on glycerol [54,55]. Another functional amino acid of ScAc2p, Arg294 (corresponding to Arg280 of ANT2) is predicted to interact with the His3 of TP4 (Figure 3E). Like Lys38 and Arg96 mutants, partial loss of function for ScAnc2p was observed in Arg294 mutants [49,52,53,56]. In ScAnc2p, the Lys38, Arg96, and Arg294 residues, together with another intrahelical Arg204, are known as the crucial amino acids required for the growth of yeast on glycerol and enzyme transport function. A molecular dynamics simulation revealed that Lys23, Arg80, and Arg280 amino acids of ANT2 allow for structural changes and functional activation of the translocator during ADP translocation [57]. Salt bridges were predicted to form between ANT2 and TP4 at Asp135-His3, Asp292-Lys14, His17-Arg21, and Glu293-Arg21/Arg24 (Figure 3E,G,H) in our model. These bridges are expected to be significant in TP4–ANT2 binding and play an important role in the stability and function of the complex. The interaction of TP4 with these crucial amino acids suggests that TP4 may inhibit conformational transitions in ANT2 and consequently disrupt adenine nucleotide translocation in mitochondria.

The hexapeptide signature (RRRMMM) is highly conserved in ANTs across species [45]. Within this motif, the Arginine triplet is essential to transporter activity due to its attraction of charged nucleotides, and no intracellular oxidative ATP production was detected in Arg252 to Arg254 (corresponding to Arg235 to 237 of ANT2) ScAnc2p mutants [49,51,52,53,56]. Another important residue in ANT2 is Tyr187, which is predicted to interact with Ile5 and Leu9 of TP4 via hydrophobic interactions (Figure 3E). Tyr187 is thought to be required for the interaction of ANT2 with ATP during the intermediate-state conformation and with the adenosine nucleotides during the internal-state conformation [58].

Overall, our study provides molecular evidence of a possible mechanism for TP4-induced damage to mitochondria, i.e., by disrupting energy metabolism through its targeting of ANT2. Furthermore, the results of our molecular TP4–ANT2 docking study may be useful for the development of TP4 mimetics to target ANT2 in diseases such as cancer.

## 4. Materials and Methods

### 4.1. Reagents and Plasmid Construction

TP4 (FIHHIIGGLFSAGKAIHRLIRRRRR) with or without biotinylation at the N-terminus was synthesized and purified by GL Biochem Ltd. (Shanghai, China), as previously described [11]. Mouse monoclonal antibody to biotin was purchased from the Santa Cruz Biotechnology (Santa Cruz, CA, USA) (clone 39-15D9). Rabbit monoclonal antibody to ANT2/SLC25A5 was purchased from Cell Signaling Technology (Boston, MA, USA) (clone E2B9D). Rabbit polyclonal antibody to TP4 was previously described [10]. Alexa Fluor-conjugated secondary antibodies were purchased from Invitrogen (Molecular Probes, Eugene, OR, USA). Rabbit immunoglobin G (IgG), horseradish peroxidase (HRP)-linked whole Ab (from donkey) secondary antibody was purchased from GE Healthcare (GE Healthcare Life Science, Buckinghamshire, UK). To generate the rabbit polyclonal antibody to monomeric Wasabi (mWasabi), full length mWasabi cDNA was amplified from the pmWasabi-C1 vector (Allele Biotechnology Inc., San Diego, CA, USA) and cloned in pBAD vector (Clontech Laboratories, Inc., Mountain View, CA). The 6×His-mWasabi protein was overexpressed in DH5α *Escherichia coli (E. coli)* and purified by Ni-affinity chromatography. The purified protein was separated by sodium dodecyl sulfate polyacrylamide gel electrophoresis (SDS-PAGE), and the gel band containing the antigen was homogenized with sterile saline, mixed with complete Freund’s adjuvant (CFA) or incomplete Freund’s adjuvant (IFA) and used for rabbit intra-dermal immunizations four times per week for two weeks. The obtained immune rabbit serum was purified by affinity chromatography using mWasabi-CNBr-sepharose 4B (GE Healthcare Life Science, Buckinghamshire, UK). To generate mWasabi-tagged ANT2, human ANT2 cDNA was amplified by PCR using specific primer pairs (Forward: XhoI-5′-CTCGAGCTATGACAGATGCCGCTGTGTCC-3′; reverse: XbaI-5′-TCTAGATATGTGTACTTCTTGATTTCATC-3′) using cDNA from MCF-7 cells as the template. The ANT2 cDNA was directly ligated into pGEM-T-easy vector (Promega Corp., Madison, WI, USA) following the manufacturer’s protocol. Sequence-verified ANT2 was then digested by XhoI and XbaI and inserted into pmWasabi-C1 that was pre-digested with the same restriction enzymes.

### 4.2. Cell Culture

Cell lines, MCF-7 and A549, were purchased from the Bioresource Collection and Research Center (BCRC). Standard culture procedures and conditions were followed, according to instructions from the BCRC. For plasmid transfection, 1 × 10^6^ A549 cells were seeded on a 10 cm^2^ plate. After overnight culture, 16 µg pmWasabi-ANT2 plasmid DNA was transfected using LipofectAMINE^TM^3000 (ThermoFisher Scientific, Carlsbad, CA, USA), according to the recommended protocol from the manufacturer. Cells were cultured for 48 h and harvested for antibody pulldown or immunocytochemical studies.

### 4.3. Immunoprecipitation and Protein Identification

Immunoprecipitation (IP) with the TP4 antibody was performed as previously described [10]. Briefly, cell lysates were first prepared in IP detergent (50 mM Tris-HCl, pH 8.0; 150 mM NaCl; 1% Igepal CA-630 (Sigma-Aldrich, St. Louis, MO, USA); protease cocktail (Roche Applied Science, Mannheim, Germany)). One milligram of protein lysate from TP4-treated A549 cells (10 µg/mL, 3 h) was incubated with primary antibody and magnetic Dynabeads (Dynabeads^TM^, Thermo Fisher Scientific, Oslo, Norway). For the LC-MS/MS analysis, boiled lysates were electrophoresed on a 12% SDS-PAGE and stained by InstantBlue^TM^ dye (Expedeon Ltd., Cambridgeshire, UK). A protein band was excised and was processed for tryptic in-gel digestion and LC-MS/MS analysis (Q-Exactive LC-MS, Thermo Scientific). Data acquired from the MS were analyzed by Mascot engine (v.2.6.0). For the pulldown assay using the mWasabi antibody, transfected cells were harvested by IP detergent. One milligram protein lysate was then incubated with mWasabi antibody and magnetic Dynabeads. Eluted protein samples from the beads were then incubated with 10 µg of TP4 overnight at 4 °C. For Western blotting, boiled samples were electrophoresed on an 8% or 15% SDS-PAGE and transferred onto polyvinylidene fluoride (PVDF) membrane. The membranes were blocked for 1 h at room temperature (RT), and incubated with primary and secondary antibodies. Membranes were visualized with enhanced chemiluminescence (Immobilon Western Chemiluminescent HRP substrate, Merck Millipore, Billerica, MA, USA) and detected with a UVP BioSpectrum^TM^ 500 imaging system (Analytik Jena AG, Thuringia, Germany).

### 4.4. Immunocytochemical and Immunohistochemical Studies

Cells were stained with Biotin and ANT2 antibodies overnight at 4 °C, followed by appropriate Alexa Fluor-conjugated secondary antibody. Hochest33258 was used for nuclear staining. For the confocal microscopic analysis, samples were mounted with fluorescent mounting medium (ProLong Gold Antifade Reagent, Thermo Fisher Scientific, Eugene, OR, USA) and images were obtained with a FV3000 laser-scanning confocal microscope (Olympus, Tokyo, Japan), using a 60× objective lens (Plapon 60×OSC2, N.A. 1.4, oil) with DAPI (EX 461, EM 359), GFP (EX 470, EM 525 for EGFP), and Cy3 (EX 550, EM 570) filter sets. Super-resolution images were taken, and deconvolution was performed using the integrated FV31S software (Olympus, Tokyo, Japan). The spatial colocalization and relative fluorescence intensities of Biotin-TP4 and ANT2 were determined by line-series analysis in ASW2.1 software. The 3D colocalization of TP4 and ANT2 was determined with Imaris software (v.9.2.1, Bitplane, Zurich, Switzerland).

### 4.5. Homology Modeling and Model Validation

The sequence of human ANT2 was retrieved from the National Center for Biotechnology Information (NCBI) protein sequence database (http://www.ncbi.nlm.nih.gov/protein) and a template (PDB ID: 1OKC; 89% identity, 99% of query coverage) was identified using PSI-BLAST [59] against the RCSB Protein Data Bank (PDB) [60]. The 3D model of ANT2 was constructed by using the standalone comparative modeling program Modeller 9.23 [61]. Twenty-five models were generated for the human ANT2, each having a different DOPE (discrete optimized protein energy) score. The protein model with the lowest DOPE score was selected for final validation. The energy minimization process of protein model was performed using GROMOS 96 43B1 force field implementation in Swiss-PDB viewer software (version 4.1) [62]. The stereochemical quality of the protein model was performed with the Ramachandran plot analysis using RAMPAGE server [63] and PROCHECK module of the PDB Sum server [64]. Root-mean-square deviation (RMSD) of the modeled structure from the selected template was calculated using RMSD calculator tool in Swiss-Pdb viewer based on the backbone atoms. Furthermore, the quality was verified using the ERRAT score values based on statistics of non-bonded atomic interactions and distribution of atoms [65], and using the ProSA analysis based on the qualitative assessment methods [66].

### 4.6. Protein–Peptide Docking

The TP4 peptide structure was retrieved from PDB as the solution structure of Tilapia Piscidin 4 (TP4) from *Oreochromis niloticus* (PDB ID: 5H2S) [67]. Structure ID: 5H2S was solved by solution NMR and contains 15 models, the authors had identified model 1 as representative, based on the criterion of lowest energy. The protein model and peptide structure were submitted to the GRAMM-X docking server [68] to perform a rigid body docking using the fast Fourier transformation (FFT) method with smoothed potentials, refinement stage, and knowledge-based scoring. The best surface match between molecules was determined by correlations using FFT. In the main input setting of GRAMM-X docking, the chain IDs of the receptor and ligand were set to A and B, respectively. Meanwhile, the number of models to save in the final output file was specified as 50. In the interface residue constraints fields, the docking to approximate location of the receptor binding site was restricted to the RRRMMM signature sequence (Arg235 to Met240). Moreover, potential interface residues of the receptor were set to 1, potential ligand interface residues were set to all, potential interface residues of the ligand were set to 1. At least one receptor–ligand contact pair had both receptor and ligand residues from the lists above. The output PDB file contained 10 models, which were ranked according to most probable prediction. Moreover, the predicted complexes were observed with protein–protein interaction by LigPlot^+^ (v.2.2) [69] one by one to select the most reasonable model. The final complex was then subjected to energy minimization with force field GROMOS 96 43B1 from Swiss-PDB viewer to perform idealization of bond geometry and removal of unfavorable non-bonded contacts. Electrostatic potential surface and interactions of the complex were calculated and visualized by using the PyMOL Molecular Graphics System (v2.0 Schrödinger, Portland, OR, USA). Peptide–protein interactions between TP4 and ANT2 were analyzed using the “Dimplot” module within the Ligplot^+^ program (v.2.2).

## Supplementary Materials

The following are available online at https://www.mdpi.com/1660-3397/18/8/417/s1. Video S1, Supplementary Data S1, Data S2, Supplementary Table S1, and Supplementary Figures S1–S4 are published online with the manuscript. Video S1: Integration of TP4 with ANT2. Three-dimensional projection was simulated with Imaris software. Data S1: ANT2 sequence identification and characterization was accomplished with a Mascot database search. Data S2: PROCHECK analyses. Structural bioinformatic and geometric analysis was used to check the stereochemical quality of the ANT2 structure. Ramachandran plots were used to analyze its overall and residue-by-residue geometry. Table S1: The Modeller log file, including a summary of all models built. For each model, assessment scores are given, according to molpdf, DOPE and GA341. Figure S1: The target-template alignment file by Modeller. All identical positions are marked with a “*”. Figure S2: Ramachandran plot values show the number of residues in favored, allowed and outlier regions. Figure S3: ERRAT diagram. The program gives a statistical analysis of the non-bonded interactions between different atom types. Lower error values indicate more favorable interactions. Figure S4: ProSA Z-score plot of modelled ANT2 protein. The Z-score for modelled ANT2 protein is represented as a black dot.
