# Supplementary material for: Antimicrobial Peptide TP4 Targets Mitochondrial Adenine Nucleotide Translocator 2
Source: Mar Drugs. 2020 Aug 9;18(8):417. doi: 10.3390/md18080417 (PMC7459631; doi:10.3390/md18080417)

MATRIX SCIENCE MASCOT Search Results

Protein View: ADT2_HUMAN

ADP/ATP translocase 2 OS=Homo sapiens GN=SLC25A5 PE=1 SV=7

**Database:**	SwissProt
**Score:**	296
**Monoisotopic mass (M_r):**	33059
**Calculated pI:**	9.71
**Taxonomy:**	Homo sapiens

Sequence similarity is available as an NCBI BLAST search of ADT2_HUMAN against nr.

Search parameters

**MS data file:**	A2018042404.mgf
**Enzyme:**	Trypsin: cuts C-term side of KR unless next residue is P.
**Fixed modifications:**	Carbamidomethyl (C)
**Variable modifications:**	Deamidated (NQ), Oxidation (M)

Protein sequence coverage: 29%

Matched peptides shown in ***bold red***.

```
  1 MTDAAVSFAK DFLAGGVAAA ISKTAVAPIE RVKLLLQVQH ASKQITADKQ
 51 YKGIIDCVVR IPKEQGVLSF WRGNLANVIR YFPTQALNFA FKDKYKQIFL
101 GGVDKRTQFW LYFAGNLASG GAAGATSLCF VYPLDFARTR LAADVGKAGA
151 EREFRGLGDC LVKIYKSDGI KGLYQGFNVS VQGIIIYRAA YFGIYDTAKG
201 MLPDPKNTHI VISWMIAQTV TAVAGLTSYP FDTVRRRMMM QSGRKGTDIM
251 YTGTLDCWRK IARDEGGKAF FKGAWSNVLR GMGGAFVLVL YDEIKKYT
```

Unformatted sequence string: 298 residues (for pasting into other applications).

Sort by ◉ residue number ○ increasing mass ○ decreasing mass
Show ◉ matched peptides only ○ predicted peptides also

Query	Start – End	Observed	Mr(expt)	Mr(calc)	ppm	M	Score	Expect	Rank	U	Peptide
12204	11 – 23	610.3377	1218.6607	1218.6608	-0.066	0	38	0.0019	1	U	K.DFLAGGVAAAISK.T
9768	24 – 31	428.7494	855.4843	855.4814	3.38	0	31	0.0068	1		K.TAVAPIER.V
13621	61 – 72	487.2721	1458.7944	1458.7983	-2.72	1	58	1.6e-005	1	U	R.IPKEQGVLSFWR.G
9769	73 – 80	428.7534	855.4923	855.4926	-0.33	0	47	0.0001	1		R.GNLANVIR.Y
8547	141 – 147	673.3874	672.3801	672.3806	-0.77	0	42	0.00068	1		R.LAADVGK.A
8548	141 – 147	673.3879	672.3806	672.3806	-0.039	0	46	0.00029	1		R.LAADVGK.A
11852	141 – 152	386.5461	1156.6164	1156.6200	-3.11	1	38	0.0024	1	U	R.LAADVGKAGAER.E
11853	141 – 152	579.3182	1156.6219	1156.6200	1.66	1	42	0.00057	1	U	R.LAADVGKAGAER.E
12622	153 – 163	431.8909	1292.6509	1292.6547	-2.97	1	31	0.014	1	U	R.EFRGLGDCLVK.I
15634	245 – 259	606.2825	1815.8258	1815.8284	-1.45	1	63	5.4e-006	1	U	R.KGTDIMYTGTLDCWR.K
15161	246 – 259	852.8666	1703.7186	1703.7284	-5.73	0	38	0.00056	1	U	K.GTDIMYTGTLDCWR.K + Oxidation (M)
9973	273 – 280	451.7442	901.4738	901.4770	-3.49	0	45	0.00053	1		K.GAWSNVLR.G

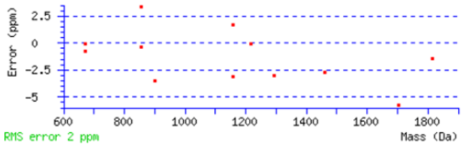

Supplement: Supplementary file 1 [file marinedrugs-18-00417-s001.zip › marinedrugs-890707 supplementary materials/Supplementary Data S1.pdf]
