# Supplementary material for: Antimicrobial Peptide TP4 Targets Mitochondrial Adenine Nucleotide Translocator 2
Source: Mar Drugs. 2020 Aug 9;18(8):417. doi: 10.3390/md18080417 (PMC7459631; doi:10.3390/md18080417)
Supplement: Supplementary file 1 [file marinedrugs-18-00417-s001.zip › marinedrugs-890707 supplementary materials/Supplementary Data S2.pdf]

Generate full PROCHECK analyses

PROCHECK summary for e857

Ramachandran plot

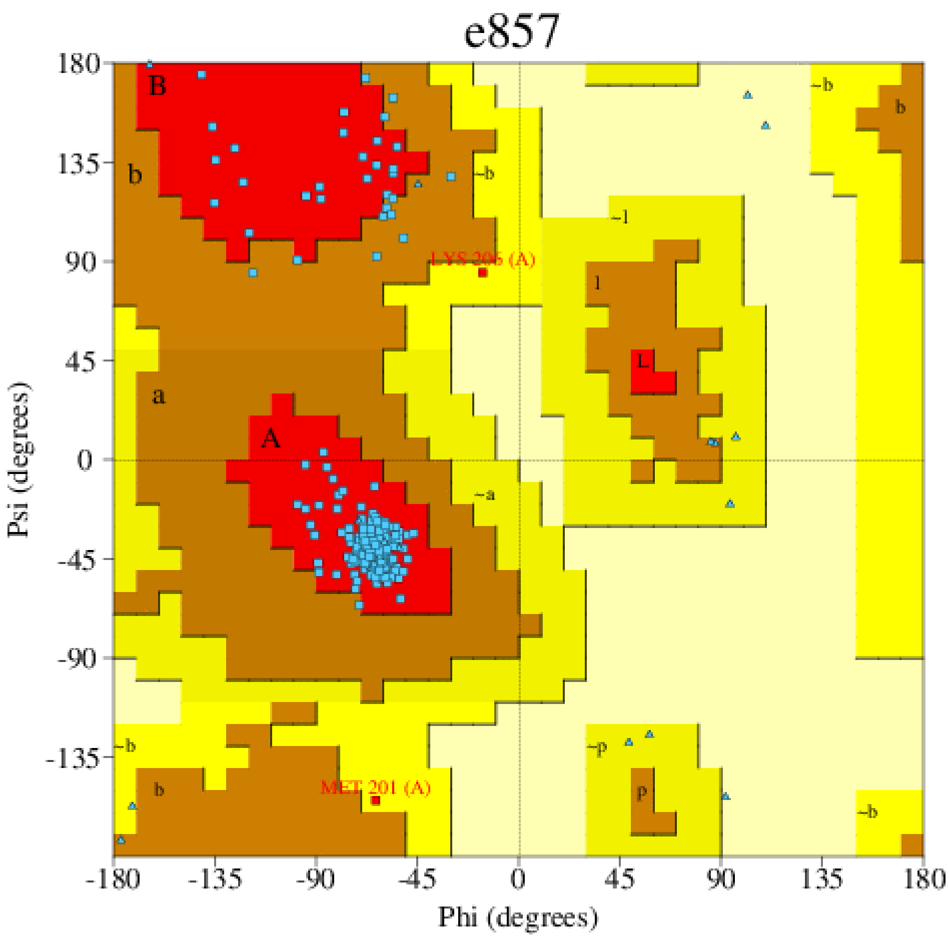

PROCHECK statistics

1. Ramachandran Plot statistics

```
                                      No. of
                                     residues     %-tage
                                      ------      ------
Most favoured regions      [A,B,L]       246       95.3%
Additional allowed regions [a,b,l,p]      10        3.9%
```

```
Generously allowed regions [~a,~b,~l,~p]      2        0.8%
Disallowed regions         [XX]               0        0.0%
                                           ----      ------
Non-glycine and non-proline residues        258      100.0%

End-residues (excl. Gly and Pro)              2

Glycine residues                             31
Proline residues                              7
                                           ----
Total number of residues                    298
```

Based on an analysis of **118** structures of resolution of at least **2.0** Angstroms and *R*-factor no greater than **20.0** a good quality model would be expected to have over **90%** in the most favoured regions [A,B,L].

2. G-Factors

```
                                                   Average
Parameter                             Score         Score
---------                             -----         -----
Dihedral angles:-
     Phi-psi distribution              0.49
     Chi1-chi2 distribution            0.09
     Chi1 only                        -0.10
     Chi3 & chi4                       0.47
     Omega                            -0.06
                                                     0.20
                                                    =====
Main-chain covalent forces:-
     Main-chain bond lengths           0.47
     Main-chain bond angles            0.19
                                                     0.30
                                                    =====

     OVERALL AVERAGE                                 0.25
                                                    =====
```

G-factors provide a measure of how **unusual**, or out-of-the-ordinary, a property is.

```
Values below -0.5*  - unusual
Values below -1.0** - highly unusual
```

Important note: The main-chain bond-lengths and bond angles are compared with the Engh & Huber (1991) ideal values derived from small-molecule data. Therefore, structures refined using different restraints may show apparently large deviations from normality.
