# Supplementary material for: Antimicrobial Peptide TP4 Targets Mitochondrial Adenine Nucleotide Translocator 2
Source: Mar Drugs. 2020 Aug 9;18(8):417. doi: 10.3390/md18080417 (PMC7459631; doi:10.3390/md18080417)
Supplement: Supplementary file 1 [file marinedrugs-18-00417-s001.zip › marinedrugs-890707 supplementary materials/Supplementary Fig. S1.pdf]

```
 _aln.pos         10        20        30        40        50        60
1OKC      --DQALSFLKDFLAGGVAAAISKTAVAPIERVKLLLQVQHASKQISAEKQYKGIIDCVVRIPKEQGFL
ant2      MTDAAVSFAKDFLAGGVAAAISKTAVAPIERVKLLLQVQHASKQITADKQYKGIIDCVVRIPKEQGVL
 _consrvd   * * ** ************************************ * ****************** *

 _aln.p   70        80        90       100       110       120       130
1OKC      SFWRGNLANVIRYFPTQALNFAFKDKYKQIFLGGVDRHKQFWRYFAGNLASGGAAGATSLCFVYPLDF
ant2      SFWRGNLANVIRYFPTQALNFAFKDKYKQIFLGGVDKRTQFWLYFAGNLASGGAAGATSLCFVYPLDF
 _consrvd ************************************   *** *************************

 _aln.pos  140       150       160       170       180       190       200
1OKC      ARTRLAADVGKGAAQREFTGLGNCITKIFKSDGLRGLYQGFNVSVQGIIIYRAAYFGVYDTAKGMLPD
ant2      ARTRLAADVGKAGAEREFRGLGDCLVKIYKSDGIKGLYQGFNVSVQGIIIYRAAYFGIYDTAKGMLPD
 _consrvd ***********  * *** *** *  ** ****  ********************** **********

 _aln.pos    210       220       230       240       250       260       270
1OKC      PKNVHIIVSWMIAQTVTAVAGLVSYPFDTVRRRMMMQSGRKGADIMYTGTVDCWRKIAKDEGPKAFFK
ant2      PKNTHIVISWMIAQTVTAVAGLTSYPFDTVRRRMMMQSGRKGTDIMYTGTLDCWRKIARDEGGKAFFK
 _consrvd *** **  ************** ******************* ******* ******* *** *****

 _aln.pos      280       290
1OKC      GAWSNVLRGMGGAFVLVLYDEI----
ant2      GAWSNVLRGMGGAFVLVLYDEIKKYT
 _consrvd **********************
```
