# Supplementary material for: Antimicrobial Peptide TP4 Targets Mitochondrial Adenine Nucleotide Translocator 2
Source: Mar Drugs. 2020 Aug 9;18(8):417. doi: 10.3390/md18080417 (PMC7459631; doi:10.3390/md18080417)
Supplement: Supplementary file 1 [file marinedrugs-18-00417-s001.zip › marinedrugs-890707 supplementary materials/Supplementary Fig. S2.pdf]

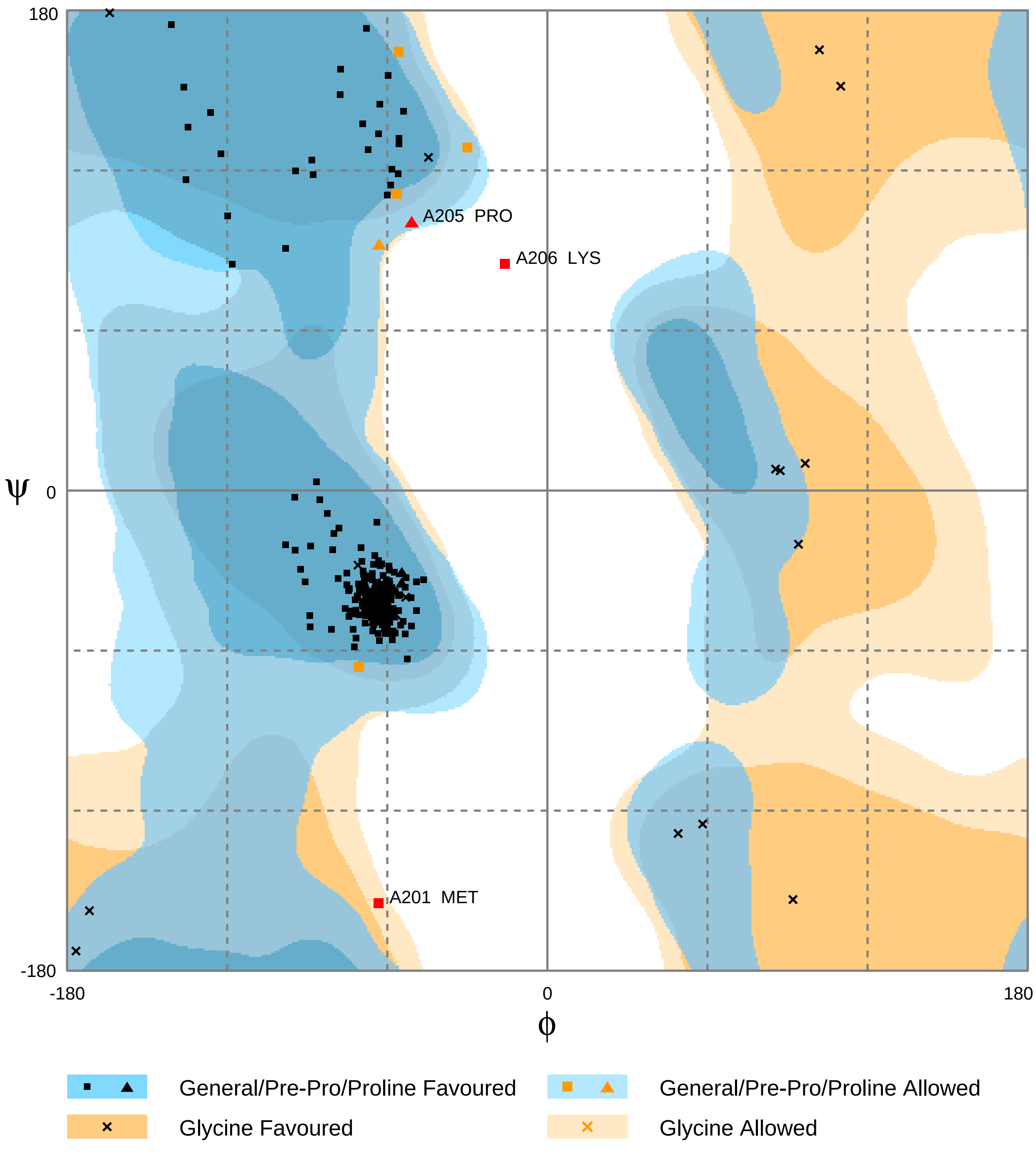

Number of residues in favoured region (~98.0% expected) : 288 (97.3%)
Number of residues in allowed region (~2.0% expected) : 5 (1.7%)
Number of residues in outlier region : 3 (1.0%)

RAMPAGE by Paul de Bakker and Simon Lovell available at http://www-cryst.bioc.cam.ac.uk/rampage/

Please cite: S.C. Lovell, I.W. Davis, W.B. Arendall III, P.I.W. de Bakker, J.M. Word, M.G. Prisant, J.S. Richardson & D.C. Richardson (2002) Structure validation by Cα geometry: φ/ψ and Cβ deviation. Proteins: Structure, Function & Genetics. **50**: 437-450

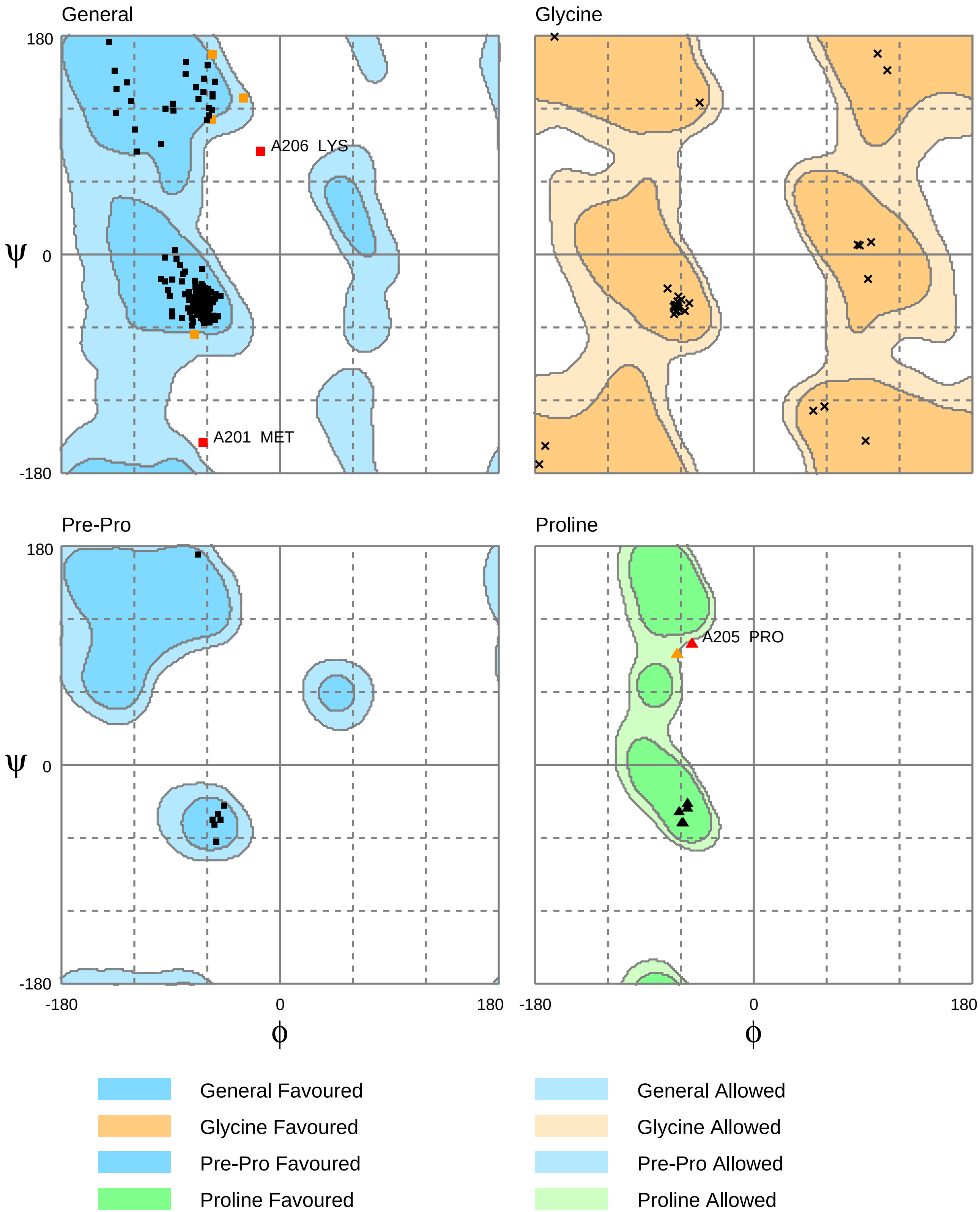

Number of residues in favoured region (~98.0% expected)	:	288 (97.3%)
Number of residues in allowed region (~2.0% expected)	:	5 (1.7%)
Number of residues in outlier region	:	3 (1.0%)

RAMPAGE by Paul de Bakker and Simon Lovell available at http://www-cryst.bioc.cam.ac.uk/rampage/

Please cite: S.C. Lovell, I.W. Davis, W.B. Arendall III, P.I.W. de Bakker, J.M. Word, M.G. Prisant, J.S. Richardson & D.C. Richardson (2002) Structure validation by Cα geometry: ϕ/ψ and Cβ deviation. Proteins: Structure, Function & Genetics. **50**: 437-450
