# Supplementary material for: Antimicrobial Peptide TP4 Targets Mitochondrial Adenine Nucleotide Translocator 2
Source: Mar Drugs. 2020 Aug 9;18(8):417. doi: 10.3390/md18080417 (PMC7459631; doi:10.3390/md18080417)
Supplement: Supplementary file 1 [file marinedrugs-18-00417-s001.zip › marinedrugs-890707 supplementary materials/Supplementary Tab. S1.pdf]

```
>> Summary of successfully produced models:
Filename                          molpdf     DOPE score    GA341 score
----------------------------------------------------------------------
ant2.B99990001.pdb            1144.87549   -31677.81055        1.00000
ant2.B99990002.pdb            1023.03369   -32158.97852        1.00000
ant2.B99990003.pdb            1062.88000   -32079.35547        1.00000
ant2.B99990004.pdb            1039.41357   -31974.30273        1.00000
ant2.B99990005.pdb            1028.65491   -32131.65430        1.00000
ant2.B99990006.pdb            1130.10791   -32094.13477        1.00000
ant2.B99990007.pdb            1060.92517   -32045.64453        1.00000
ant2.B99990008.pdb            1043.07666   -31908.79297        1.00000
ant2.B99990009.pdb            1114.37866   -31994.77344        1.00000
ant2.B99990010.pdb            1091.30322   -31639.03711        1.00000
ant2.B99990011.pdb            1130.36694   -31793.13672        1.00000
ant2.B99990012.pdb            1051.51685   -32166.15430        1.00000
ant2.B99990013.pdb             987.78619   -32094.70117        1.00000
ant2.B99990014.pdb            1032.18799   -32102.73047        1.00000
ant2.B99990015.pdb            1044.15063   -32347.17773        1.00000
ant2.B99990016.pdb            1213.12744   -31967.70313        1.00000
ant2.B99990017.pdb            1036.52454   -32105.75391        1.00000
ant2.B99990018.pdb            1028.71826   -32066.68164        1.00000
ant2.B99990019.pdb            1301.35095   -31753.09180        1.00000
ant2.B99990020.pdb            1075.85803   -32169.12305        1.00000
ant2.B99990021.pdb             997.95160   -32268.30859        1.00000
ant2.B99990022.pdb            1160.16650   -32141.12891        1.00000
ant2.B99990023.pdb             963.28333   -32145.35938        1.00000
ant2.B99990024.pdb            1007.97034   -32246.53906        1.00000
ant2.B99990025.pdb            1004.13037   -31975.49414        1.00000
```
