# Supplementary figures and images for: Antimicrobial Peptide TP4 Targets Mitochondrial Adenine Nucleotide Translocator 2
Source: Mar Drugs. 2020 Aug 9;18(8):417. doi: 10.3390/md18080417 (PMC7459631; doi:10.3390/md18080417)

# Results for ant2-model.pdb, chain A (298 aa)

Overall model quality

[HELP](#)

Z-Score: **-4.78**

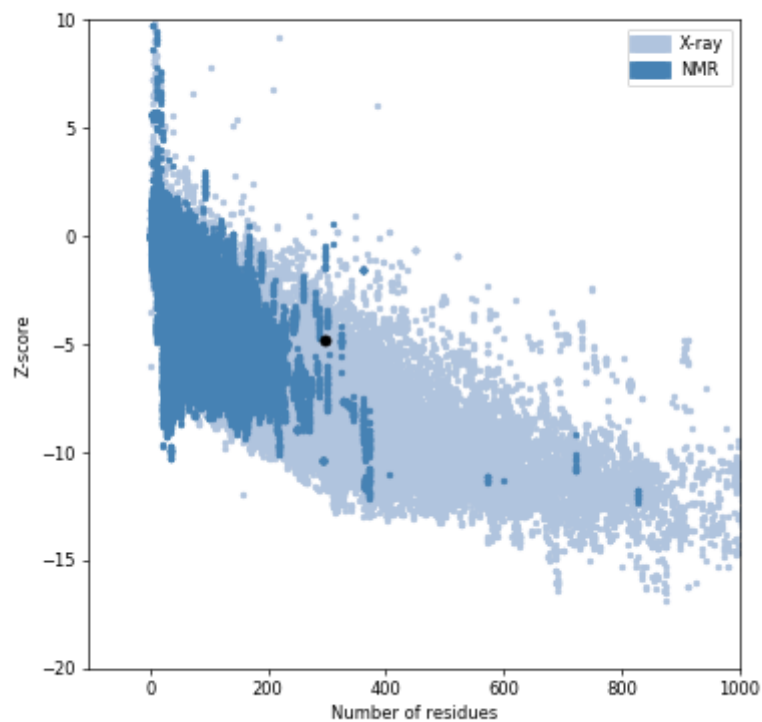

Supplement: Supplementary file 1 [file marinedrugs-18-00417-s001.zip › marinedrugs-890707 supplementary materials/Supplementary Fig. S4.pdf]
